# Radiologic findings that aid in the reduction of misdiagnoses of Langerhans cell histiocytosis of the bone: a retrospective study

**DOI:** 10.1186/s12957-021-02261-y

**Published:** 2021-05-10

**Authors:** Mimi Zhao, Limin Tang, Shiqing Sun, Jiufa Cui, Haisong Chen

**Affiliations:** Department of Radiology, The Affiliated Hospital of Qingdao University, Qingdao, 266003 China

**Keywords:** Langerhans cell histiocytosis, Radiology, Differential, Diagnostic imaging, Bone lesion

## Abstract

**Background:**

This study aimed to identify the characteristic radiological signs for the diagnosis of Langerhans cell histiocytosis (LCH) of the bone.

**Methods:**

We retrospectively studied 82 cases of LCH with bone lesions confirmed by pathology. Clinical and radiological features of the patients were analyzed.

**Results:**

A total of 64 and 18 patients had single and multiple bone lesions, respectively. With regard to LCH with single bone lesions, 37.5% (24/64) of lesions were located in the skull and presented as bone destruction with or without soft tissue mass. The correct diagnosis rate of these lesions was 60.0% (9/15) in children and adolescents, but was only 22.2% (2/9) in adults.

A total of 26.5% (17/64) of the solitary lesions were found in the spine. Of these, 88.2% (15/17) were located in the vertebral body and appeared to have different degrees of collapse, and 66.7% (10/15) of these lesions were correctly diagnosed.

Of the unifocal lesions, 21.8% (14/64) were located in other flat and irregular bones and manifested as osteolysis. Only 21.4% (3/14) of these cases were correctly diagnosed.

In total, 14.1% (9/64) of the isolated bone LCH lesions were located in the long bones. Of these, 77.8% (7/9) were located in the diaphysis and presented as central bone destruction with or without fusiform periosteal reaction and extensive peripheral edema, of which 42.9% (3/7) were correctly diagnosed before surgery or biopsy.

With regard to LCH with multiple bony destructive lesions, 71.4% (10/14) of cases in children and adolescents were correctly diagnosed; however, all four cases among adults were misdiagnosed.

**Conclusion:**

In all age groups, isolated diaphyseal destruction of the long bone with fusiform periosteal reaction and extensive peripheral edema, vertebra plana of the spine, and bevelled edge of skull defects accompanied by soft tissue masses strongly suggest LCH diagnosis. Moreover, the multiple bone osteolytic destruction in children and adolescents strongly suggests LCH diagnosis. Familiarity with these typical radiological signs of LCH is necessary to decrease misdiagnoses.

## Background

Histiocytoses are rare disorders characterized by the accumulation of macrophages, dendritic cells, and monocyte-derived cells in various tissues and organs. Langerhans cell histiocytosis (LCH) was assigned to the Langerhans-associated disease (L) group [1]. LCH, the most common histiocytic disorder, is characterized by the accumulation of CD1A+/CD207+ mononuclear phagocytes within granulomatous lesions that can affect nearly all organ systems. Based on genetic, molecular, and functional data, LCH is defined as an inflammatory myeloid neoplasia [2].

LCH is categorized as a single-system LCH (SS-LCH) with multifocal or unifocal involvement, or as multisystem LCH (MS-LCH) with multiple organ involvement with or without risk organ involvement. Organs at risk include the hematopoietic system (bone marrow), spleen, and liver [3]. LCH may affect any organs, but those more frequently affected are the bones (80% of cases), skin (33%), pituitary gland (25%), liver, spleen, hematopoietic system, lungs (15% each), lymph nodes (5–10%), or the central nervous system (CNS) (2–4%, excluding the pituitary) [4]. The diagnosis of LCH is based on histological and immunophenotypic examinations. The primary indicators are characteristic LCH cells and positivity for CD1a and/or Langerin (CD207) cells. Confirmation of cytoplasmic Birbeck granules by electron microscopy is no longer necessary [5]. Radiological measures, including computed tomography (CT) and magnetic resonance imaging (MRI), are also recommended to evaluate LCH and assist in diagnosis [6].

Although the skeletal system may be involved in 80% of LCH cases, the diagnosis of bone LCH by medical imaging remains challenging. Our study retrospectively evaluated 82 cases of LCH patients with bone lesions to identify the characteristic radiological signs for the correct diagnosis of LCH of the bone.

## Methods

### Patients

Patients with LCH confirmed by pathology who had undergone diagnostic imaging in our hospital were included in this study. A systematic computerized search of the hospital database was performed to identify eligible patients under the diagnostic code of LCH who were admitted between January 2008 and June 2020. The inclusion criteria were as follows: (1) patients with pathologically confirmed LCH; (2) patients who underwent X-ray, CT, or MRI examination prior to the initiation of surgical treatment; and (3) patients with bone lesions observed on imaging and had available imaging data.

Informed consent was obtained from all of the patients or their guardians. This study was approved by the ethics committee of our hospital. A total of 82 patients were included, with 64 and 18 patients having single and multiple bone lesions, respectively. Of all cases, 49 patients were male and 33 were female (male to female ratio=1.5:1). The demographics of the participants are shown in Table 1.

**Table 1:** The demographic of patients with Langerhans cell histiocytosis with bone lesions

	Single	Multiple
Patients (No.)	64	18
Sex (M/F)	38/26	11/7
Median age at diagnosis (Y)	13.5 (1–64)	4.5 (1–66)
Age distribution (No. [%])		
Child (range, 1–17)	37 (57.8)	14 (77.8)
Adult (range, 19–66)	27 (42.2)	4 (22.2)

*No.* number of patients

### Image acquisition

Thirty minutes before the CT or MR scan, 5% chloral hydrate (1 mL/kg) was orally administered to sedate the children when necessary.

X-rays were performed using the Philips DR system (Netherlands) and the Digital Diagnost Version workstation.

CT scans were performed using a GE (US) 128-slice CT scanner (Discovery CT 750HD) and Siemens (Germany) 64-slice CT machine. The scanning parameters were as follows: slice thickness, 0.6 mm; tube voltage, 120–340 keV; tube current, 80–240 mAs; and pitch, 1. After scanning, multiplanar reconstruction was performed to obtain the sagittal and coronal images. CT images with window levels of 250–350 Hu and window widths of 1000–1500 Hu were used to observe bone structures; CT images with window levels of 40–60 Hu and window widths of 300–500 Hu were used to observe soft tissues.

MR was performed using a US GE Signa 1.5T and GE HDx 3.0T superconducting magnetic resonance scanner. The scanning matrix was 256×192, the layer thickness was 4 mm, and the scanning sequence and its parameters were set as follows: SE T1WI (TR 400–600 ms, TE 5–25 ms); FSE T2WI (TR 3000–4000 ms, TE 80–105 ms), and short-time inversion recovery sequences (STIR) or T2WI pre-saturated fat suppression sequences (TR 3000–4000 ms, TE 80–105 ms). Post-contrast scans were performed with fat-suppressed T1-weighted imaging (TR 400–600 ms, TE 5–25 ms) after intravenous injection of 0.2 mg/kg gadolinium-diethylenetriamine pentaacetic acid (Gd-DTPA, Magnevist, Bayer-Schering Pharma AG).

### Image analysis

Two experienced skeletal radiologists who were blinded to the results of other tests, including pathological diagnosis, evaluated the images separately. The location, number, and imaging characteristics of the lesions were recorded. If the two radiologists disagreed, a consensus was reached through discussion.

The patients were followed-up every month for the first 3 months, every 3 months for the first year, annually for the next 2 years, and biennially thereafter. Radiological examination at follow-up included radiography, CT, or MR examination.

### Statistical analyses

The incidence of LCH lesions at different sites and the rate of preoperative misdiagnosis were compared. Continuous data were summarized as medians and ranges, and categorical data were calculated using frequency counts and percentages. The diagnostic consistency of the two radiologists was calculated using the kappa value. The strength of agreement was considered good for values between 0.76 and 1.00.

## Results

### Imaging modalities and diagnosis

X-ray, CT, and MR images were available for 29 patients, and the correct diagnosis rate of these cases was 51.7% (15/29). X-ray and CT studies were performed for another 53 patients and the correct diagnosis rate of these cases was 41.5% (22/53). The correct diagnosis rate did not differ between groups (*P*>0.05).

### Lesion distribution and symptoms

The two evaluators agreed on the outcomes of all patient image evaluations and exhibited good consistency (*K* = 0.90).

The distribution and number of skeletal lesions among the patients are shown in Table 2–a total of 172 lesions. A total of 64 patients had single bone lesions. On the other hand, 18 patients had multiple lesions, with 108 total lesions among these 18 cases.

**Table 2:** Localization and number of skeletal lesions among patients with Langerhans cell histiocytosis

Localization	Single lesion			Multiple lesions			Total (%)
	Child	Adult	Total (%)	Child	Adult	Total (%)	
Skull	15	9	24 (37.5)	30	0	30 (27.8)	54 (31.4)
Spine	7	10	17 (26.6)	23	10	33 (30.6)	50 (29.1)
Long bones	9	0	9 (14.1)	13	0	13 (12.0)	22 (12.8)
Rib	4	8	12 (18.7)	5	5	10 (9.3)	22 (12.8)
Pelvic bone	1	0	1 (1.6)	13	2	15 (13.9)	16 (9.3)
Others	1	0	1 (1.6)	5	2	7 (6.5)	8 (4.7)
Total	37	27	64 (100)	89	19	108 (100)	172 (100)

Others: includes the clavicle, scapula, and sternum

The clinical symptoms of LCH patients are shown in Table 3.

**Table 3:** The clinical symptoms of patients with Langerhans cell histiocytosis with skeletal lesions

Clinical symptoms	Single lesion				Multiple lesions
	Skull	Long bones	Spine	Others	
Pain	14	7	16	13	9
Soft tissue mass	7	0	0	0	4
Ear drainage	2	0	0	0	0
Polydipsia/polyuria	1	0	0	0	0
Soft tissue welling	1	1	1	0	2
Restricted movement	0	1	0	0	1
Fever	0	0	0	0	2
No symptoms	0	0	0	1	0

Others: includes the pelvis, ribs, clavicle, scapula, and sternum

### Radiographic findings and diagnosis

Regarding LCH with single bone lesions, 37.5% (24/64) of lesions were located in the skull/cranial-facial bone. These lesions presented as bone destruction (100%, 24/24), bevelled edge (50.0%, 12/24), soft tissue mass (79.2%, 19/24), and the correct diagnosis rate of these lesions was 60.0% (9/15) among children and adolescents, but only 22.2% (2/9) in adults.

Seventeen patients (26.5%, 17/64) had isolated spine bone LCH lesions. Of these, 88.2% (15/17) were located in the vertebral body and appeared as different degrees of collapse (100%, 15/15), vertebral plana (33.3%,5/15), obvious surrounding marrow and soft tissue edema (60%, 9/15), and soft-tissue extension (53.3%, 8/15), with 66.7% (10/15) being correctly diagnosed. Of these, 11.8% (2/17) only involved the vertebral arch and/or posterior elements, which were misdiagnosed as metastatic tumors or infectious diseases, respectively.

Nine patients (14.1%, 9/64) had isolated long-bone LCH lesions. Of these, 77.8% (7/9) affected the diaphysis and presented as the central bone destruction. Other manifestations included fusiform periosteal reaction (71.4%, 5/7) and extensive surrounding marrow and soft tissue edema (57.1%, 4/7), with 42.9% (3/7) being correctly diagnosed before the surgical procedure or biopsy. Of these, 22.2% (2/9) were located in the metaphysis or epiphysis and presented as the bone destruction involving the epiphyseal plate. All of these were misdiagnosed as osteoblastomas and infectious diseases, respectively.

In total, 21.8% (14/64) of the isolated lesions of LCH were located in other flat and irregular bones (including the mandible, pelvis, rib bone, etc.). These lesions manifested as osteolysis (100%, 14/14), soft tissue mass (35.7%,5/14), soft tissue edema (14.3%, 2/14), and pathological bone (21.4%, 3/14), with only 21.4% (3/14) being correctly diagnosed.

Multiple sites were involved among the 18 LCH patients presenting with multiple bony destruction. Of the cases among children and adolescents, 71.4% (10/14) were correctly diagnosed; however, the four adults were all misdiagnosed (0/4). Three of them were misdiagnosed with metastatic tumours, and one case was misdiagnosed as an infectious disease.

Among the LCH cases included in this study, 55 were treated in our hospital and had radiographic follow-up data. The treatment strategies were varied: 31 patients underwent surgery alone, 10 patients underwent surgery followed by chemotherapy, 13 patients received chemotherapy as the only treatment modality, and one patient did not receive any treatment. The mean follow-up duration for the 55 patients who received treatment averaged 27.6 months (range, 6–100 months). During the follow-up period, the primary lesions of 53 patients decreased or disappeared, and no new lesions appeared. Two or 3 months after treatment, sclerotic edges were observed in the lesions of four patients who received chemotherapy. Local recurrence of LCH was observed in only two children (3.6%, 2/55). In one patient, the primary lesion was located in the temporal bone. However, a new lesion of the iliac bone was found 3 months after surgery during follow-up CT. Another patient was diagnosed with MS-LCH with multifocal and involved risk organs (bone marrow and liver). Three months after chemotherapy, this patient’s CT showed an increase in the number of lesions.

## Discussion

LCH may occur at any age, but it is mainly observed among children and adolescents [7, 8]. In our series of studies, children accounted for 62.2% (51/82) of the patients. Additionally, several studies have reported a male preponderance, citing ratios between 1.2 and 2.0 to 1 [9–11]. In our study population, the male to female ratio was 1.5:1, which is roughly similar to previously reported ratios. The findings of Reisi., et al. [12] revealed that the unifocal form was the most common form of bone involvement, which is similar to our findings. The clinical manifestations of LCH are diverse, and the most common clinical manifestation of patients with bone involvement is soft tissue mass accompanied by swelling or pain [4], which is consistent with our group. LCH can affect any bone, but it is more likely to occur in the skull, affecting 45–50% of patients [9, 13]. In our series, the skull (31.4%,54/172) was most commonly affected, with the lesions mostly located in the calvaria (59.3%, 32/54), followed by the maxillofacial and skull base bones, which is roughly similar to the findings in a previous study [9]. In the present study, 44.0% of the spine lesions (22/50) were located in the thoracic vertebrae, followed by the lumbar and cervical vertebrae, which is consistent with the findings of Bertram et al. [14]. We also found that the long bone LCH lesions were mainly located in the femur (10/22, 45.5%), which is consistent with previous reports [15, 16]. Long bone LCH reportedly affects mainly the metaphysis or diaphysis, but does not affect the epiphysis [17, 18]; in the present study, two patients were observed to have LCH involvement in the epiphysis. Reisi., et al. [12] reported that the sternum, clavicle, scapula, and humerus were rare lesion locations. Similarly, they were rare in our study, with only 1 to 2 cases observed in each.

**Fig. 1:**
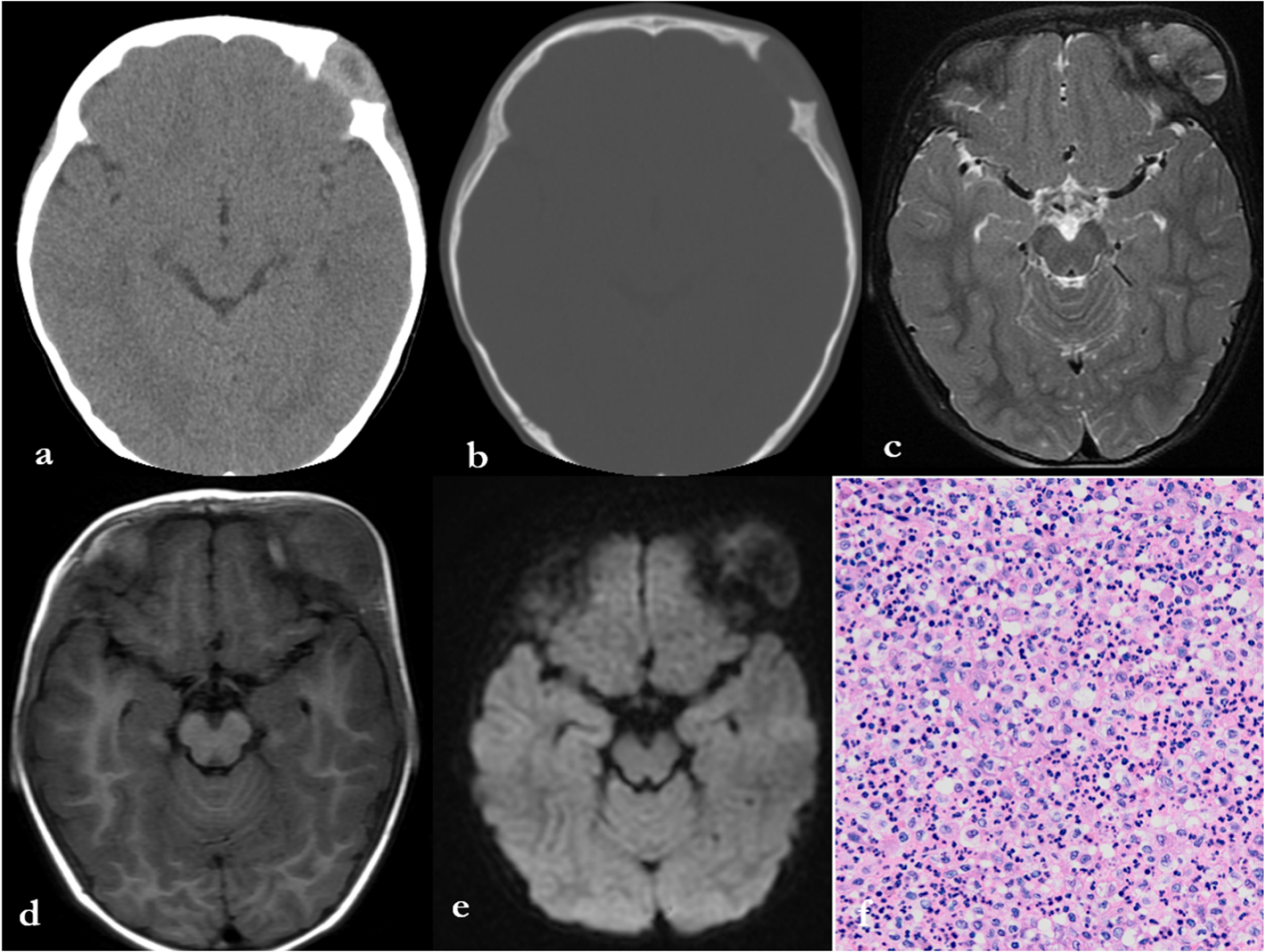
Axial computed tomography of a 2-year-old male patient (**a**, **b**) showing bevelled-edge appearance in the left frontal bone. Axial MR images show hyperintensity on T2WI (**c**), hypointensity on T1WI (**d**), and slight hypointensity on diffusion weighted imaging (**e**). The diagnosis of LCH was confirmed by pathology after surgery (pathological slice HE × 200) (**f**)

The typical imaging findings of LCH single cranial lesions are mainly round or oval bone defects, which are usually lytic, with clear boundaries (Fig. 1). Due to the asymmetrical destruction of the inner and outer plates, the LCH skull lesions appear to be “punched out.” The destruction of the inner plate is usually greater than that of the outer plate, and bevelled edges can be observed [9]. As reported in a previous article, cases with characteristic bevelled edges are not common. However, 50% (12/24) of the patients in our single cranial group exhibited this feature, all occurring in the calvarium, which may be due to the high proportion of calvarial lesions. Bone destruction may also be related to soft tissue masses [17]. A total of 79.2% of the lesions (19/24) in our group were accompanied by soft tissue masses. However, the “bevelled edges” are not unique to LCH. Myelomas and bone epidermoid cysts can also exhibit this feature [19]. However, myeloma is often accompanied by osteoporosis. Lytic lesions of the skull are also typical presentations of osseous epidermoid cysts, but the inner table is generally more affected than the outer table [20]. Skull destruction of metastatic tumors may also be accompanied by soft tissue masses, which are difficult to distinguish, especially in adults [13]. In our study, the rate of correct diagnosis in children is much higher than that in adults, possibly because radiologists rarely consider metastatic tumors and myeloma in the differential diagnosis of pediatric patients.

**Fig. 2:**
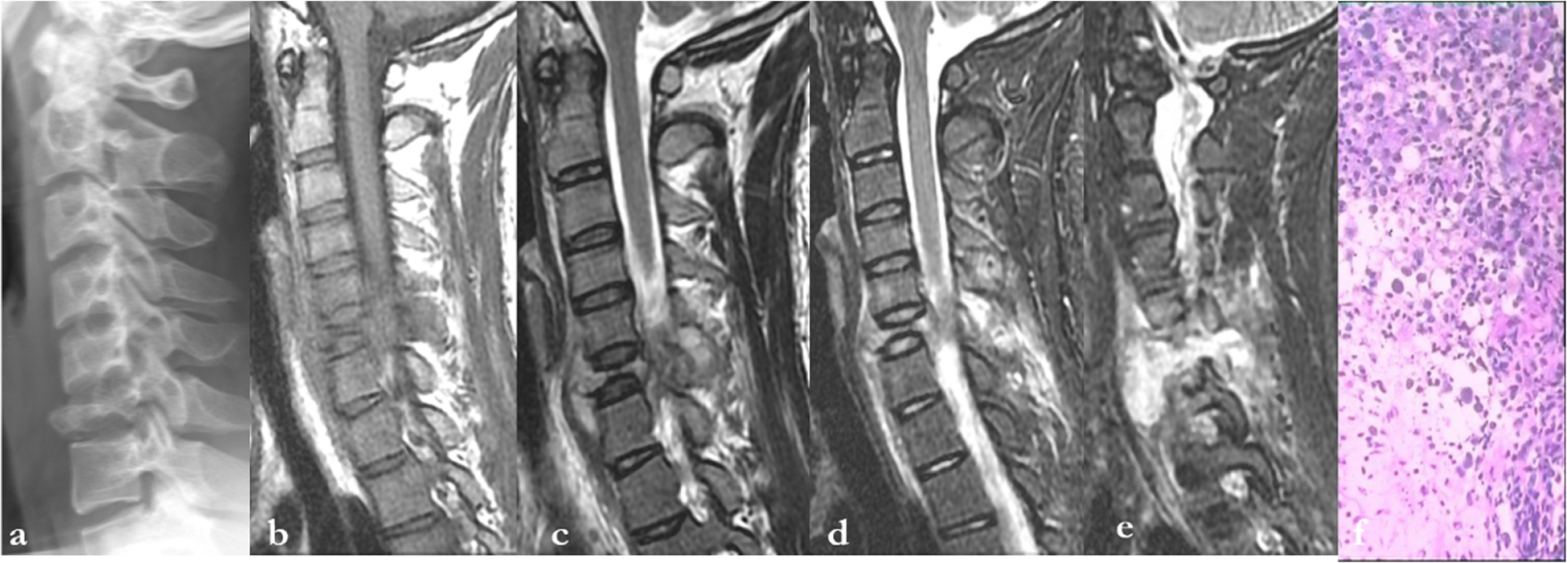
Spinal X-ray and sagittal MR image of the cervical spine of a 19-year-old male patient with neck pain and restricted neck motion. X-ray (**a**) shows a lesion in the C6 associated with severe vertebral collapse. The lesion shows slight hypointensity on T1WI (**b**), hyperintensity on T2WI (**c**), and hyperintensity on fat-saturated T2WI (**d**, **e**), associated with obvious marrow and soft tissue edema, and prevertebral and epidural soft tissue extension. The diagnosis of LCH was confirmed by pathology after surgery (pathological slice HE × 200) (**f**)

The imaging features of typical spinal LCH lesions reported in the literature mainly include complete or incomplete collapse of the vertebral body, retention of the intervertebral disc space, and associated paravertebral soft tissue masses (Fig. 2). The vertebral plana is often observed in paediatric patients, and the diseased vertebral body may completely flatten into a coin shape [21]. In our study, typical vertebra plana accounted for only 33.3% (5/15) of cases in the vertebral body group, all of which were children. As described in the literature, the performance of the flat vertebrae is relatively specific but rare [22]. All patients with vertebral plana in our study were diagnosed correctly. With the popularity of CT and MRI, soft tissue extension has been reported in 50% of cases [23]. In our series of studies, soft tissue extension was observed in 53.3% of lesions (8/15). Edema of the soft tissues around the vertebral body has rarely been reported in the literature, but we found it to be relatively common. In our group, 60% (9/15) of the lesions had obvious edema in the surrounding soft tissues. Moreover, spine LCH lesions can extend to paravertebral soft tissues, and even involve the intervertebral disc space or endplate, which may be confused with malignant tumors such as metastatic tumors and myeloma, especially in older patients.

The typical imaging findings of LCH flat and irregular bone lesions reported in the literature are mainly dilated or lytic bone destruction. Rib lesions are often accompanied by pathological fractures, and soft tissue extension may cause extrapleural masses [24, 25]. In our series, only 21.4% (3/14) of the cases were associated with pathological fractures, and 35.7% (5/14) were associated with soft tissue masses. No specific diagnostic signs were identified, and the diagnostic accuracy was very low.

**Fig. 3:**
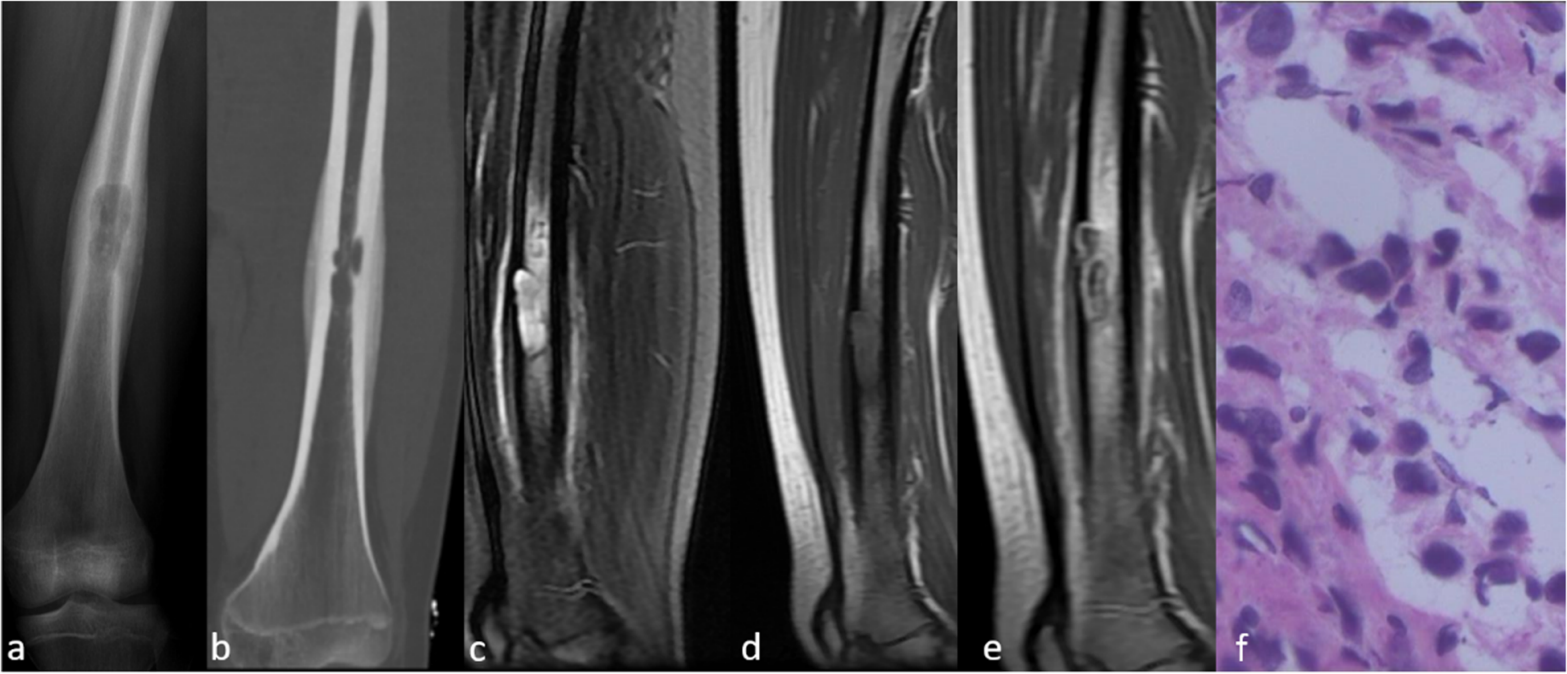
X-ray (**a**), CT (**b**), and MRI (**c**-**e**) of the left femur of a 12-year-old female patient. X-ray image (**a**) and CT (**b**) show an oval lesion in the middle left femoral diaphysis, associated with fusiform periosteal reaction. The lesion shows hyperintense signals and surrounding marrow and soft tissue oedema on FS-T2WI (**c**), hypointense signal on T1WI (**d**), and obvious enhancement after gadolinium administration. The diagnosis of LCH was confirmed by pathology after surgery (pathological slice HE × 400) (**f**)

The typical imaging findings of LCH long bone lesions reported in the literature are mainly round or oval clear radiolucent areas, which are mostly cystic or expansive bone destruction (Fig. 3) [26]. Periosteal reactions are common and vary in size, with characteristic multi-layer onion skin or single-layer morphology [27]. In our study, most of the lesions exhibited fusiform multilayer periosteal reactions. Moreover, long bone LCH is usually accompanied by extensive periosteal reaction, which needs to be distinguished from long bone malignancies such as Ewing’s sarcoma. One of the differential points is that most LCH lesions are surrounded by obvious marrow and soft tissue edema due to the inflammatory medium released by LCH; conversely, the peritumoral edema of the malignancy is relatively small due to the immune escape of the malignant tumors [28]. On the other hand, LCH that occurs at the metaphysis is difficult to distinguish from other diseases such as osteoblastoma and tuberculosis of the bone. In our study, both patients with lesions in the metaphysis were misdiagnosed.

In our cohort, the patients with multiple bone involvement sites presented with multiple bone destruction. LCH with multiple bone destruction lesions had a higher diagnostic accuracy rate in children (71.4%, 10/14), but had been difficult to be correctly diagnosed in adults. With regard to LCH with multiple bone destruction lesions, no adult patient in our group was correctly diagnosed (0/4). Three of them were older than 45 years and were misdiagnosed with metastatic tumors; among them, one patient had a history of prostate cancer. Another case of a young male was misdiagnosed as having an infectious disease due to the involvement of several adjacent vertebral bodies, which was very similar to infectious lesions. Because diseases associated with multiple bone lesions other than LCH are rare in children, radiologists should prioritise LCH in the differential diagnosis of pediatric patients.

The prognosis of LCH also varies depending on the form of the disease (SS-LCH vs. MS-LCH), as well as its location and response to chemotherapy. In unifocal LCH involving a bone or isolated skin lesion, the prognosis is good. Spontaneous remission or symptoms that subsided after local treatment have been reported [3]. In forms with multifocal bone involvement, disease relapse occurs more often [5]. By contrast, the recurrence rate was low among our patients (3.6%). This may be related to the patients we selected; in the present study, most cases had a single bone involvement. On the other hand, the emergence of marginal sclerosis has been reported as an indicator of mature and localised lesions [16]. However, it is rarely observed. This is consistent with our findings. At first, we observed sclerosis on the edge of the lesions in the follow-up CT images of four patients, with the lesions eventually becoming reduced or subsided and with no new lesions observed.

In general, among LCH patients with bone lesions, children are more likely to be diagnosed correctly than adults. In the present study, more than half of the children (56.9%, 29/51) were correctly diagnosed by a radiologist. Solitary skull and spine lesions (for patients with single bone lesions) and multiple bone lesions in young patients have a high probability of being correctly diagnosed as LCH. The typical locations and imaging signs of LCH bone lesions increases the correct diagnostic rate of the disease by a radiologist.

Nevertheless, this study has some limitations. The retrospective design of the study means that there may be some confusion and bias. Non-uniform imaging techniques are also a limitation of our research, as not all patients had available X-ray, CT, and MR images of the bones. However, in our cohort, the diagnostic accuracy did not differ between groups of patients with different imaging modalities (*P*>0.05). This indicates that the imaging modality of patients in this study did not influence diagnostic accuracy. One possible explanation for this is that most of the typical radiological signs of LCH (except for surrounding marrow edema) can be observed simultaneously on CT and MR. Finally, the study is also limited by its small sample size. Therefore, a multi-center study is required to evaluate more cases.

## Conclusions

In most cases, the imaging findings of LCH with bone lesions are non-specific and can vary according to the location of the lesion and the disease progression. However, in all age groups, isolated diaphyseal destruction of the long bone with fusiform periosteal reaction and peripheral oedema, vertebra plana of the spine, and bevelled edge of the skull defects accompanied by soft tissue masses strongly suggest the diagnosis of LCH. Moreover, the multiple bone osteolytic destruction in children and adolescents strongly suggests the diagnosis of LCH. Familiarity with these typical radiological signs of LCH can decrease misdiagnoses.

## Data Availability

All data generated or analyzed during the study were included in this article.

## Abbreviations

CT: Computed tomography
LCH: Langerhans cell histiocytosis
SS-LCH: Single-system LCH
MS-LCH: Multisystem LCH
MR: Magnetic resonance
STIR: Short-time inversion recovery sequences
